# Supplementary material for: Coevolution of vocal signal characteristics and hearing sensitivity in forest mammals
Source: Nat Commun. 2019 Jun 25;10:2778. doi: 10.1038/s41467-019-10768-y (PMC6592901; doi:10.1038/s41467-019-10768-y)
Supplement: Supplementary file 1 — Supplementary Information [file 41467_2019_10768_MOESM1_ESM.pdf]

Supplementary Information

Coevolution of vocal signal characteristics and hearing sensitivity in forest mammals

Charlton et al.

This PDF file includes:

Supplementary Methods
Supplementary Figures 1-3
Supplementary Tables 1-8
Supplementary References

Supplementary Methods

GSUPraat tools1.7/quantifyFFT.praat

The "quantifyFFT.praat" script was written by Michael J. Owren and is freely available as part of GSU Praat Tools 1.7. The script computes a Fast Fourier Transform (FFT) spectrum for each of the sound files. For our analysis we used the procedure 'objects_nolabels", used the entire sound file (rather windowing relative to peak amplitude or select time points), and choose an FFT size of 1024. The procedure works as follows:

1) The sampling frequency and period of the sound file are extracted and used to set the analysis window length in milliseconds. The sampling period for a sound file sampled at 40 kHz (40,000 samples per second) is 0.000025 seconds (1 sample is taken every 0.000025 seconds = 40,000 per second).
2) An FFT spectrum is computed. We used an FFT size of 1024, giving a frequency bin width of 19.5 Hz (frequency bandwidth of 20000/1024 = 19.5).
3) The frequency bin numbers and amplitude in Pascal are saved as a matrix, and Pascal is converted to decibel (dB) values.
4) The maximum dB value is used to rescale the spectrum to a normalised range that matches the number of frequency values.
5) The spectral slope is calculated as the slope of a linear regression line fit to the dB values of each frequency bin.

Supplementary Figure 1. The phylogeny used to control for shared ancestry between 51 different terrestrial mammal species in the phylogenetic generalized least-squares regressions that tested the effect of habitat on hearing sensitivity measures (taken from ref. [1]).

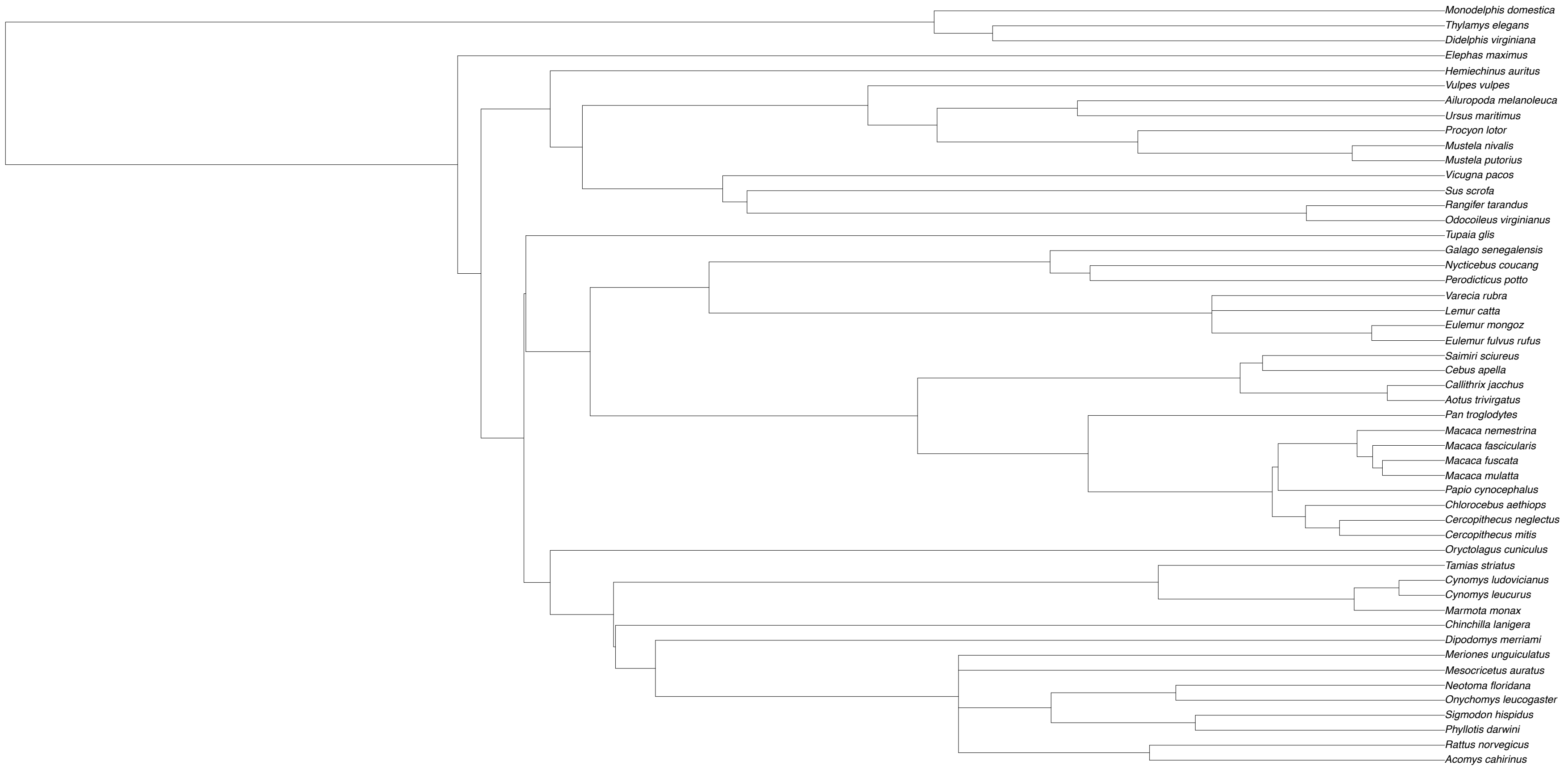

Supplementary Figure 2. The phylogeny used to control for shared ancestry between 116 different terrestrial mammal species in the phylogenetic generalized least-squares regressions that tested the effect of habitat on spectral slope (taken from[1]).

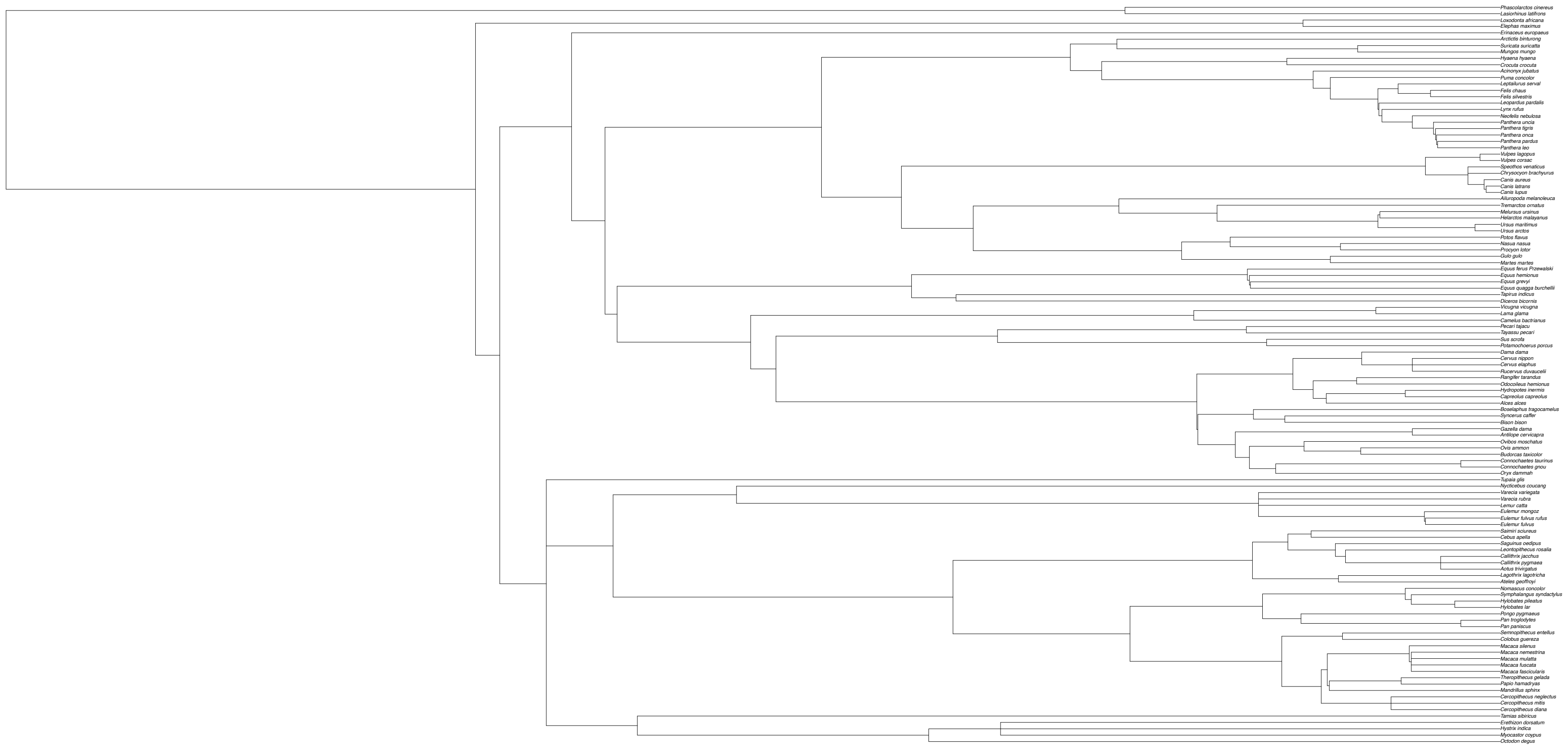

Supplementary Figure 3. The phylogeny used to control for shared ancestry between 17 different terrestrial mammal species in the phylogenetic generalized least-squares regressions that tested the effect of spectral slope on hearing sensitivity measures (taken from ref. [1]).

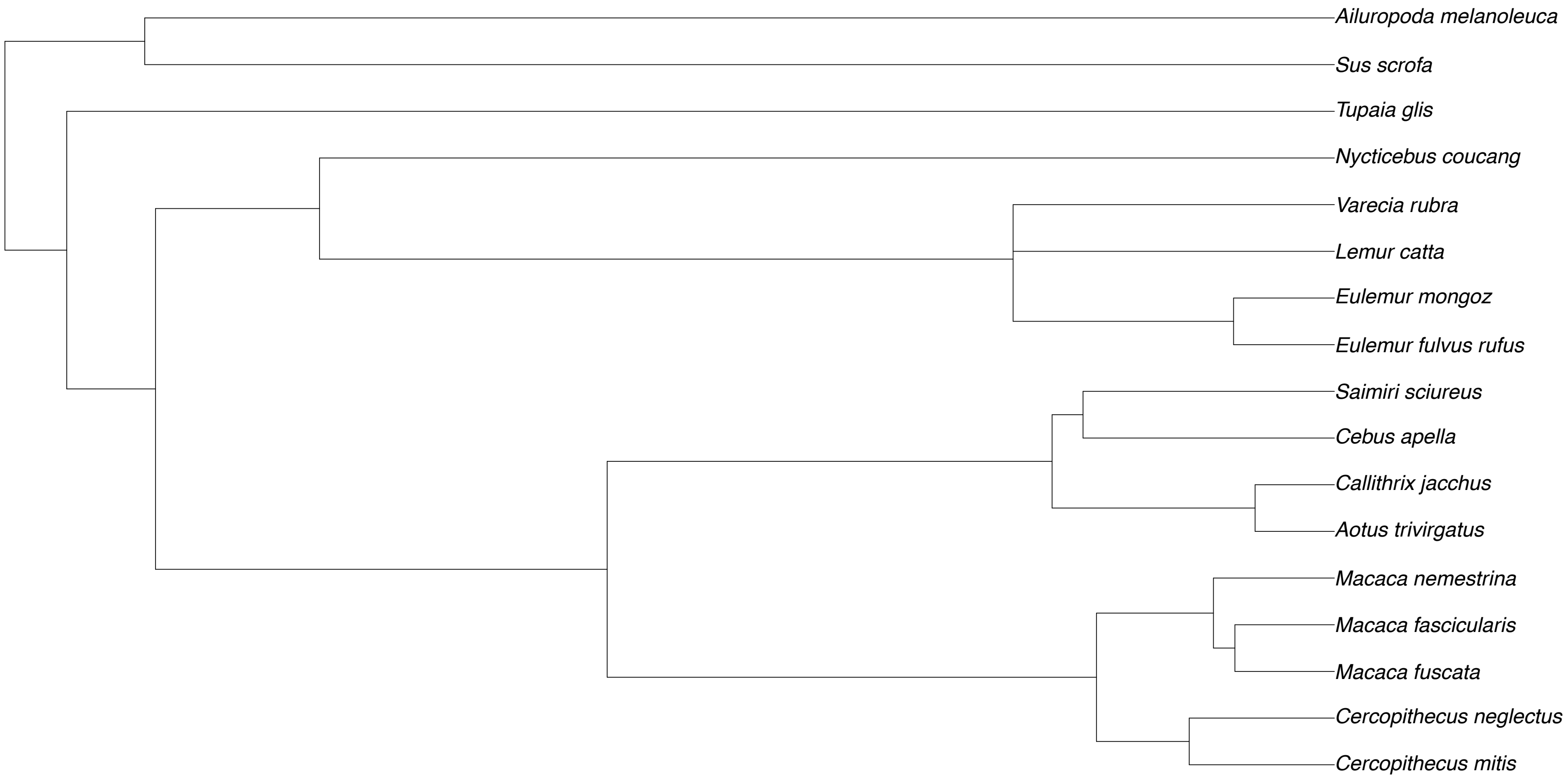

Supplementary Table 1. Audiogram data for the 51 terrestrial mammal species in the comparative analysis.

Species	Functional head size (µs)	Peak sensitivity (kHz)	Mean sensitivity (dB)	Mean sensitivity 10-20 kHz (dB)	Relative high frequency hearing sensitivity	Source of audiogram data
Acomys cahirinus	64	8	37	19.8	17.2	2
Ailuropoda melanoleuca	583[#]	12.5	22.2	10.6	11.6	3
Aotus trivirgatus	225	10	9.5	6	3.5	4
Callithrix jacchus	170	7	21.9	21.5	0.4	5
Cebus apella[$]	414[#]	12*	34.4	34.7	-0.3	6
Cercopithecus mitis	400	4	14.4	9.3	5.1	7,8
Cercopithecus neglectus	420	5.7	17.5	8.8	8.7	9
Chinchilla lanigera	225	1	25.3	26.6	-1.3	10
Chlorocebus pygerythrus	400	1.4	15.5	10.1	5.4	9
Cynomys leucurus	120	8	35.4	39	-3.6	11
Cynomys ludovicianus	134	4	44.3	38.1	6.2	*11*
Didelphis virginiana	273	20	34.1	18.8	15.3	12,13
Dipodomys merriami	90	1	21.3	15.8	5.5	14,15
Elephas maximus	3350	1	50.7	86	-35.3	16
Eulemur fulvus rufus[$]	237[#]	11.3	20.8	13.4	7.4	17
Eulemur mongoz[$]	240[#]	8	22	18.4	3.6	17
Galago senegalensis	212	8	17.9	0.9	17	18
Hemiechinus auritus	167	8	27.8	9	18.8	19
Lemur catta	280	8	15.5	12.4	3.1	20
Macaca fascicularis	410	16	17.1	15.6	1.5	21,22
Macaca fuscata	465	4	25.2	7.8	17.4	23
Macaca mulatta	424	16	13.6	9.9	3.7	21,22

Macaca nemestrina	470	4.5*	16.3	15.3	1.0	[24]
Marmota monax	290	4	32.2	35.1	-2.9	[2]
Meriones unguiculatus	87	3*	12.6	6.5	6.1	[25]
Mesocricetus auratus	114	10	30.1	16.4	13.7	[2]
Monodelphis domestica	114	16	42.2	24.1	18.1	[26]
Mustela nivalis	76	2	25	1	24	[27]
Mustela putorius	180	12	23.8	8.3	15.5	[28]
Neotoma floridana	115	8	25.4	7	18.4	[29]
Nycticebus coucang	218	16	27.9	10.9	17	[30]
Odocoileus virginianus	801	8	27	12.8	14.2	[31]
Onychomys leucogaster	61	8	34.1	18.3	15.8	[29]
Oryctolagus cuniculus	250	2	22.5	8.1	14.4	[15]
Pan troglodytes	650	8	14.7	16.5	-1.8	[32]
Papio cynocephalus	580	8	11	6.5	4.5	[33]
Perodicticus potto	218	16	20.4	2.6	17.8	[30]
Phyllotis darwini	64	11	26.1	14.3	11.8	[2]
Procyon lotor	320	1	1.6	-3.5	5.1	[34]
Rangifer tarandus	548	8	20.2	7.7	12.5	[35]
Rattus norvegicus	150	8	23.8	9	14.8	[36]
Saimiri sciureus	302	10	19.5	16.3	3.2	[37,38]
Sigmodon hispidus	65	8	22	1.3	20.7	[2]
Sus scrofa	498	8	23.1	13.6	9.5	[39]
Tamias striatus	108	1	41.8	21.1	20.7	[2]
Thylamys elegans	60	8	57.8	43.5	14.3	[26]
Tupaia glis	136	16	19.1	-3	22.1	[40]
Ursus maritimus	583$^{\#}$	6.5	18	22	-4	[41]

Varecia rubra[$]	317[#]	11.3	23.6	14.7	8.9	[17]
Vicugna pacos	544	8	24.1	15.1	9	[42]
Vulpes vulpes	219[#]	4	-0.5	7.5	-8	[43]

Functional head size data taken from http://www.utoledo.edu/al/psychology/research/psychobio/audiograms2.html; *Frequency of best sensitivity taken as average of two frequency values with the same threshold; [#]Functional head size estimated from skull width; [$]Auditory Brainstem Response (ABR) method

Supplementary Table 2. Acoustic data for the 116 terrestrial mammal species in the comparative analysis.

Species	N (Ind)	N (calls)	Gender	Spectral slope	Source of audio recordings	Description of call types	Behavioral context of call production
Acinonyx jubatus	3	30	♂♀	-0.55	1	Stutter barks, growls, squeals	Various[44]
Ailuropoda melanoleuca	15	75	♂♀	-0.46	3	Bleats, chirps	Contact[45]
Alces alces	4	55	♂♀	-0.43	2, 4	Moans, grunts	Advertisement[46]
Antilope cervicapra	3	23	♂♀	-0.52	1	Grunts	Alarm[47]
Aotus trivirgatus	2	16	Unknown	-0.39	6	Tonal alarm calls	Alarm#
Arctictis binturong	2	11	♂♀	-0.30	1	Trills, chirps, defense calls	Various#
Ateles geoffroyi	4	66	Unknown	-0.50	1	Squeals, squeaks, screams	Various[48]
Bison bison	4	15	♂♀	-0.45	1, 2	Bellows	Advertisement[49]
Boselaphus tragocamelus	2	9	♂	-0.5	1	Moan	Aggression[50]
Budorcas taxicolor	2	10	♂♀	-0.44	1	Pulsed grunts	Advertisement[51]
Callithrix jacchus	3	35	♂♀	-0.25	1	Phees, trills, chitters	Various[48]
Callithrix pygmaea	2	13	Unknown	-0.06	1	Trills	Contact[52]
Camelus bactrianus	3	39	♂♀	-0.42	1	Hum, roars, squeals	Various[53]
Canis aureus	3	19	♂♀	-0.38	1	Howls, squeals	Various[54]
Canis latrans	2	27	Unknown	-0.44	2	Barks, howls, growls	Various[55]
Canis lupus	4	35	♂♀	-0.36	1, 2	Howls, growls	Aggression[54]

Capreolus capreolus	3	16	♂♀	-0.47	4	Barks	Advertisement[56]
Cebus apella	5	38	Unknown	-0.4	1	Trills, chirps	Various[57]
Cercopithecus diana	2	10	Unknown	-0.38	1	Trills, alert calls	Contact[58]
Cercopithecus mitis	2	15	Unknown	-0.40	5	Pyows	Disturbance[59]
Cercopithecus neglectus	3	20	♂♀	-0.50	1	Keckers	Alarm[60]
Cervus elaphus	4	30	♂	-0.46	4	Harsh and common roars	Advertisement[46]
Cervus nippon	4	11	♂	-0.48	1	Moans	Advertisement[46]
Chrysocyon brachyurus	4	15	♂♀	-0.56	1	Barks	Disturbance[61]
Colobus guereza	3	28	♂	-0.52	1	Roars	Advertisement[62]
Connochaetes gnou	3	28	♂	-0.57	1	Barks, honks	Various[53]
Connochaetes taurinus	3	18	♂♀	-0.46	1	Grunts	Contact[53]
Crocuta crocuta	2	14	♂	-0.38	1, 2	Whoops	Group coordination[63]
Dama dama	5	34	♂♀	-0.5	1	Groans, moans	Advertisement[46]
Diceros bicornis	3	20	Unknown	-0.52	1	Mating calls, moans	Various[64]
Elephas maximus	2	34	♂♀	-0.49	1	Trumpets, rumbles, growls	Various[65]
Equus ferus Przewalski	5	15	♂♀	-0.48	1	Brays	Contact[66]
Equus grevyi	3	10	♂	-0.48	1	Brays	Various[67]
Equus hemionus	3	35	♂♀	-0.59	1	Brays, moans, defense calls	Various[53]
Equus quagga burchellii	2	25	♂	-0.46	1	Brays, barks, honks	Various[53]
Erethizon dorsata	2	22	♀	-0.48	1	Whines	Aggression[68]
Erinaceus europaeus	2	13	Unknown	-0.52	1	Keckers, squawks	Various[69]
Eulemur fulvus	2	16	Unknown	-0.46	1	Alarm calls	Alarm#
Eulemur fulvus rufus	5	105	♂♀	-0.37	6	Alarm calls	Alarm#
Eulemur mongoz	2	20	Unknown	-0.40	1	Keckers, alarm calls	Alarm#
Felis chaus	3	56	♂♀	-0.46	1	Mews, yowls, growls	Various[70]
Felis silvestris	4	24	♂♀	-0.43	1, 2	Long calls, mews	Contact[70]

Gazella dama	2	11	♂♀	-0.42	1	Pulsed grunts	Advertisement[71]
Gulo gulo	2	18	♀	-0.53	1	Growls, snarls	Aggression[72]
Helarctos malayanus	3	53	♂♀	-0.46	1	Moans, growls, barks, keckers	Various#
Hyaena hyaena	2	12	Unknown	-0.55	1	Snarls, approach calls	Various#
Hydropotes inermis	2	31	♂	-0.58	1	Whistle, bark	Various[46]
Hylobates lar	2	41	♂♀	-0.38	1	Song	Various[73]
Hylobates pileatus	2	23	♂	-0.42	1	Song	Advertisement[74]
Hystrix indica	2	23	Unknown	-0.55	1	Loud coughs, snorts	Various#
Lagothrix lagotricha	2	13	♀	-0.29	1	Trills, eeolks	Various[75]
Lama glama	2	29	♂	-0.52	1, 2	Gurgles, moans	Advertisement#
Lasiorhinus latifrons	8	134	♀	-0.49	3	Harsh coughs	Disturbance[76]
Lemur catta	2	11	♂	-0.49	1	Chirps, moans, wail	Various[77]
Leontopithecus rosalia	2	20	♂♀	-0.48	1	Trills, long call	Group coordination[78]
Leopardus pardalis	3	25	♂	-0.42	1, 2	Growls, meows	Various[79]
Leptailurus serval	2	36	♂♀	-0.57	1	Yowls	Aggression[79]
Loxodonta africana	2	22	♀	-0.44	2	Trumpets, rumbles	Various[80]
Lynx rufus	3	23	♂♀	-0.41	1, 2	Growls, , yowls, mews	Various[81]
Macaca fascicularis	2	15	Unknown	-0.51	1	Screams, trills	Various[82]
Macaca fuscata	2	21	♀	-0.43	1	Screams, shrieks,	Aggression[83]
Macaca mulatta	2	14	♀	-0.55	1	Aggressive calls	Aggression#
Macaca nemestrina	2	13	♀	-0.1	1	Moans, chirps, chitters	Various[84]
Macaca silenus	3	27	♂	-0.46	1	Grunts, whistles	Various[85]
Mandrillus sphinx	3	10	♂♀	-0.55	1	Grunts and whoops	Contact[86]
Martes martes	3	17	♂♀	-0.46	1	Growls, screeches	Aggression#
Melursus ursinus	2	17	♂♀	-0.49	1	Roar, howls, grunts	Various[87]
Mungos mungo	4	13	♂♀	-0.47	1	Trills	Group coordination[88]

Myocastor coypus	2	18	Unknown	-0.46	1	Moans, whines	Various[89]
Nasua nasua	3	17	♂♀	-0.16	1	Screeches, squeaks, chirps	Various[90]
Neofelis nebulosa	5	25	♂♀	-0.47	1, 2	Roar, growls, meows, churr	Various[79]
Nomascus concolor	2	31	♂♀	-0.41	1	Song	Various[91]
Nycticebus coucang	2	10	Unknown	-0.55	1	Defense calls	Aggression#
Octodon degus	2	83	♀	-0.29	1	Chirps, squeaks, squawks	Various[92]
Odocoileus hemionus	4	62	♂♀	-0.52	1, 2	Grunts, pulsed calls, wails	Various[53]
Oryx dammah	3	20	♂♀	-0.39	1	Pulsed moos, aggression call	Aggression#
Ovibos moschatus	2	10	♂♀	-0.4	1	Groans, roars	Advertisement[93]
Ovis ammon	5	36	♂♀	-0.53	1	Bleats	Contact[53]
Pan paniscus	3	24	♂♀	-0.24	1	Screams	Aggression[94]
Pan troglodytes	3	34	♂♀	-0.41	1, 2	Screams, pants, hoots	Various[95]
Panthera leo	6	83	♂♀	-0.39	1, 2	Roars, growls	Various[79]
Panthera onca	4	65	♂♀	-0.47	1	Roars, growls	Various[79]
Panthera pardus	4	33	♂♀	-0.39	1, 2	Roars, growls, wails	Various[79]
Panthera tigris	5	65	♂♀	-0.41	1, 2	Roars, growls	Various[79]
Panthera uncia	3	23	Unknown	-0.44	1, 2	Roars, growls, defense calls	Various[79]
Papio hamadryas	3	21	♂	-0.49	1	Grunts, shrill bark	Various[96]
Pecari tajacu	2	14	♀	-0.4	1	Low grunt, screams	Various[97]
Phascolarctos cinereus	20	70	♂♀	-0.38	3	Bellows	Advertisement[98]
Pongo pygmaeus	5	61	♂♀	-0.43	1, 2	Long calls, screams, grunts	Various[99]
Potamochoerus porcus	2	16	♂♀	-0.38	1	Grunts, groan, squeals	Various[100]
Potos flavus	2	13	♀	-0.41	1	Squeaks	Aggression[101]
Procyon lotor	4	59	Unknown	-0.53	1, 2	Chitters, keckers, growls, snarls	Various[102]
Puma concolor	5	61	♂♀	-0.48	1, 2	Roars, snarls, growls, wails	Various[79]
Rangifer tarandus	3	31	♂♀	-0.56	1, 4	Grunts	Contact[53]

Rucervus duvaucelii	2	12	♂	-0.51	1	Alarm calls	Alarm#
Saguinus oedipus	2	16	Unknown	-0.41	1	Chirps, whistles	Various[103]
Saimiri sciureus	5	56	Unknown	-0.36	1	Twitters, squeals, chirps	Various[104]
Semnopithecus entellus	2	21	♀	-0.39	1	Twitters, honk, chirps	Various[105]
Speothos venaticus	2	37	♂♀	-0.57	1	Whines, yips	Various[61]
Suricata suricatta	11	41	♂♀	-0.5	3	Barks	Alarm[106]
Sus scrofa	4	75	♀	-0.51	1, 2	Grunts	Various[53]
Symphalangus syndactylus	2	71	♂♀	-0.41	1	Song	Various[107]
Syncerus caffer	2	21	♂♀	-0.52	1	Pulsed grunts	Aggression[108]
Tamias sibiricus	2	22	Unknown	-0.51	1	Chips, Chucks	Various[109]
Tapirus indicus	3	19	♂♀	-0.36	1	Squeaks and whistles	Various#
Tayassu pecari	2	15	♂	-0.5	1	Grunts	Various[53]
Theropithecus gelada	2	27	♀	-0.5	1	Moans, grunts	Various[110]
Tremarctos ornatus	2	80	♂♀	-0.38	1	Moans, barks	Various#
Tupaia glis	2	27	Unknown	-0.58	1	Chatters, chirps	Disturbance[111]
Ursus arctos	2	27	Unknown	-0.39	1, 2	Roars, moans	Various[112]
Ursus maritimus	4	75	♂♀	-0.41	1, 2	Roars, moans	Various#
Varecia rubra	2	24	Unknown	-0.62	1	Keckers, alarm calls	Alarm#
Varecia variegata	4	28	♂	-0.47	1	Squawk, Roar/Shriek, chatter, whine	Various[113]
Vicugna vicugna	2	13	♂	-0.61	1	Whistles, chirps, alarm calls	Various#
Vulpes corsac	6	34	♂♀	-0.51	1	Barks, moans, keckers, growls	Various[114]
Vulpes lagopus	2	26	♂♀	-0.45	1	Barks, howls	Various[54]

# - behavioural context from audio recording metadata.
1 - Downloaded from the Animal Sound Archive at the Museum für Naturkunde Berlin (http://www.animalsoundarchive.org/)
2 - Animal Trax audio CD (Hollywood Edge, CA, USA)
3 - First author's personal collection of animal recordings
4 - "Des Vois Dans la Fore^t. Un Guide Sonore des Cervides d'Europe" audio CD (Sittelle, Mens. 1999)

5 - Animal calls of Africa. Gillard Bird Cassettes. South Africa (1985)
6 – Macaulay library at the Cornell Lab of Ornithology

Supplementary Table 3. Body weight and habitat data for each of the terrestrial mammal species in the dataset

Species	Mass (g)	Natural habitat	Forest	Data sources
Acinonyx jubatus	48500	Desert, grassland, savanna, scrubland	No	[115,116]
Acomys cahirinus	55	Scrubland, rocky areas	No	[115,117]
Addax nasomaculatus	92500	Desert, grassland, savanna	No	[115,118]
Ailuropoda melanoleuca	135000	Forest	Yes	[119,120]
Alces alces	290250	Forest, wetlands,	Yes	[115,121]
Antilope cervicapra	36700	Grassland, desert, forest	No	[115,122]
Aotus trivirgatus	800	Forest	Yes	[123,124]
Arctictis binturong	13000	Forest	Yes	[125,126]
Ateles geoffroyi	7535	Forest	Yes	[123,127]
Bison bison	569625	Scrubland, savanna, desert, grassland, forest	No	[115,128]
Boselaphus tragocamelus	194500	Grassland, scrubland, forest	No	[115,129]
Budorcas taxicolor	271913	Scrubland, grassland, forest	No	[125,130]
Callithrix jacchus	361.5	Forest, savanna	Yes	[115,131]
Callithrix pygmaea	116	Forest	Yes	[123,132]
Camelus bactrianus	495000^	Desert	No	[133]
Canis aureus	9380	Forest, grassland, scrubland, savanna,	No	[115,134]
Canis latrans	13394	Forest, desert, scrubland, grassland	No	[115,135]
Canis lupus	42850	Forest, desert, rocky areas, scrubland, grassland, wetlands	No	[115,136]
Capreolus capreolus	32616	Forest, grassland, scrubland	No	[115,137]
Cebus apella	2910	Forest, savanna	Yes	[115,138]

Cercopithecus diana	4550	Forest	Yes	[123,139]
Cercopithecus mitis	7110	Forest	Yes	[115,140]
Cercopithecus neglectus	5480	Forest	Yes	[115,141]
Cervus elaphus	129517	Rocky areas, forest, grassland, scrubland,	No	[115,142]
Cervus nippon	33500	Grassland, forest, scrubland,	No	[115,143]
Chinchilla lanigera	417	Rocky areas, scrubland	No	[144,145]
Chlorocebus aethiops	4807	Scrubland, savanna	No	[115,146]
Chrysocyon brachyurus	23250	Forest, wetlands, grassland, scrubland, savanna,	No	[115,147]
Colobus guereza	11350	Forest	Yes	[123,148]
Connochaetes gnou	132250	Grassland, scrubland	No	[125,149]
Connochaetes taurinus	190250	Grassland, savanna	No	[115,150]
Crocuta crocuta	66519	Grassland, savanna, forest	No	[115,151]
Cynomys leucurus	909	Grassland, scrubland	No	[115,152]
Cynomys ludovicianus	743	Grassland, savanna, desert	No	[115,153]
Dama dama	52900	Scrubland, forest, grassland	No	[115,154]
Diceros bicornis	868000	Desert, scrubland, savanna	No	[115,155]
Didelphis virginiana	2195	Forest, scrubland	Yes	[115,156]
Dipodomys merriami	38	Scrubland, desert	No	[115,157]
Elephas maximus	3755000	Scrubland, forest, grassland	No	[125,158]
Equus quagga burchellii	384200	Scrubland, grassland, savanna	No	[125,159]
Equus ferus Przewalski	999600	Desert, grassland	No	[125,160]
Equus grevyi	221000	Grassland, scrubland	No	[115,161]
Equus hemionus	286129	Grassland, desert, scrubland, savanna	No	[125,162]
Erethizon dorsatum	7593	Grassland, forest, scrubland	No	[125,163]
Erinaceus europaeus	912	Grassland, forest,	No	[115,164]

Eulemur fulvus	205	Forest	Yes	[123,165]
Eulemur fulvus rufus	222	Forest	Yes	[123,165]
Eulemur macaco	244	Forest	Yes	[123,166]
Eulemur mongoz	149	Forest	Yes	[123,167]
Felis chaus	6560	Forest, wetlands, desert, grassland, scrubland, savanna	No	[115,168]
Felis silvestris	3554	Forest, desert, scrubland, savanna, grassland	No	[115,169]
Galago senegalensis	156	Forest, savanna	Yes	[115,170]
Gazella dama	55000	Desert, savanna, scrubland	No	[115,171]
Geomys bursarius	193	Grassland, savanna	No	[115,172]
Gulo gulo	12700	Rocky areas, mountain, scrubland, forest, grassland	No	[119,173]
Helarctos malayanus	57500	Forest, scrubland	Yes	[125,174]
Hemiechinus auritus	500	Scrubland, desert	No	[19,175]
Hyaena hyaena	37463	Wetlands, savanna, scrubland, forest, grassland	No	[115,176]
Hydropotes inermis	12850	Wetlands, Marine intertidal, scrubland, forest, grassland	No	[115,177]
Hylobates lar	5620	Forest	Yes	[123,178]
Hylobates pileatus	5470	Forest	Yes	[74,123]
Hystrix indica	15675	Scrubland, grassland, forest,	No	[115,179]
Lagothrix lagotricha	6300	Forest, savanna	Yes	[115,180]
Lama glama	142500	Scrubland, grassland	No	[181,182]
Lasiorhinus latifrons	28500	Grassland, savanna, forest	No	[125,183]
Lemur catta	2210	Forest, scrubland	Yes	[123,165]
Leontopithecus rosalia	508	Forest	Yes	[115,184]
Leopardus pardalis	11900	Savanna, scrubland, forest	No	[115,185]
Leptailurus serval	10589	Grassland, wetlands, forest, savanna	No	[115,186]
Loxodonta africana	4035000	Wetlands, forest, grassland, scrubland, savanna, desert	No	[125,187]

Lynx rufus	9384	Desert, scrubland, savanna, forest, grassland	No	[115,188]
Macaca fascicularis	4125	Forest, wetlands	Yes	[115,189]
Macaca fuscata	13425	Forest	Yes	[115,190]
Macaca mulatta	6591	Scrubland savanna, forest,	No	[115,191]
Macaca nemestrina	8255	Forest	Yes	[115,192]
Macaca silenus	6280	Forest	Yes	[115,193]
Mandrillus sphinx	18250	Forest	Yes	[115,194]
Marmota monax	3780	Grassland, forest	No	[115,195]
Martes martes	1146	Forest, scrubland	Yes	[115,196]
Melursus ursinus	97500	Scrubland, grassland, forest, savanna	No	[115,197]
Meriones unguiculatus	56	Grassland, scrubland, desert	No	[115,198]
Mesocricetus auratus	88	Grassland	No	[125,199]
Monodelphis domestica	106	Forest, grassland, scrubland,	Yes	[115,200]
Mungos mungo	1187	Savanna, forest, scrubland, grassland	No	[115,201]
Mustela nivalis	82	Wetlands, forest, scrubland, rocky areas, grassland	No	[115,202]
Mustela putorius	950	Wetlands, grassland, forest, scrubland	No	[115,203]
Myocastor coypus	6824	Wetlands	No	[115,204]
Nasua nasua	3863	Forest, scrubland	Yes	[115,205]
Neofelis nebulosa	10725	Forest, scrubland	Yes	[115,206]
Neotoma floridana	255	Forest, grassland, scrubland, rocky areas, wetlands	No	[115,207]
Nomascus concolor	7705	Forest	Yes	[123,208]
Nycticebus coucang	910	Forest	Yes	[115,209]
Octodon degus	202	Scrubland	No	[125,210]
Odocoileus hemionus	49232	Scrubland, desert, wetlands, grassland, forest, marine intertidal, savanna	No	[115,211]
Odocoileus virginianus	66516	Wetlands, savanna, forest, desert, grassland, scrubland, marine	No	[115,212]

		intertidal		
Onychomys leucogaster	28	Desert, grassland, scrubland	No	[115,213]
Oryctolagus cuniculus	1528	Scrubland, forest, grassland, savanna	No	[115,214]
Oryx dammah	145250	Scrubland, desert, savanna	No	[115,215]
Ovibos moschatus	290833	Grassland	No	[125,216]
Ovis ammon	96450	Scrubland, forest, rocky areas, grassland, desert	No	[125,217]
Pan paniscus	39100	Forest	Yes	[123,218]
Pan troglodytes	44875	Forest, savanna	No	[115,219]
Panthera leo	163417	Savanna scrubland grassland forest	No	[115,220]
Panthera onca	80950	Forest, scrubland, wetlands, savanna, grassland	No	[119,221]
Panthera pardus	37450	Scrubland, savanna, grassland, desert, forest	No	[115,222]
Panthera tigris	175000	Forest, scrubland, grassland	No	[115,223]
Panthera uncia	32500	Grassland, Rocky areas, scrubland, forest	No	[119,224]
Papio cynocephalus	15163	Forest, scrubland, savanna	Yes	[115,225]
Papio hamadryas	14940	Scrubland, grassland, rocky areas	No	[125,226]
Pecari tajacu	20000	Forest, scrubland, savanna, desert, grassland	No	[115,227]
Perodicticus potto	1230	Forest	Yes	[123,228]
Phascolarctos cinereus	6583	Forest	Yes	[115,229]
Phyllotis darwini	41	Scrubland	No	[2,230]
Pongo pygmaeus	56600	Forest	Yes	[115,231]
Potamochoerus porcus	60500	Forest	Yes	[115,232]
Potos flavus	1635	Forest	Yes	[115,233]
Procyon lotor	7508	Forest	Yes	[115,234]
Puma concolor	57325	Forest, desert, grassland, savanna, scrubland	No	[115,235]
Rangifer tarandus	117250	Grassland, forest	No	[125,236]

Rucervus duvaucelii	176500	Savanna, grassland, wetlands, forest	No	[115,237]
Saguinus oedipus	364	Forest	Yes	[115,238]
Saimiri sciureus	747	Forest	Yes	[115,239]
Semnopithecus entellus	15088	Forest, scrubland, savanna	Yes	[115,240]
Sigmodon hispidus	93	Desert, grassland	No	[115,241]
Speothos venaticus	8000	Scrubland, forest, grassland, savanna	No	[119,242]
Suricata suricatta	745	Scrubland, savanna, grassland, desert	No	[115,243]
Sus scrofa	80525	Wetlands, forest, grassland, savanna, desert, scrubland	No	[115,244]
Symphalangus syndactylus	10725	Forest	Yes	[115,245]
Syncerus caffer	521542	Scrubland, grassland, wetlands, forest, savanna	No	[125,246]
Tamias sibiricus	93	Forest, scrubland	Yes	[125,247]
Tamias striatus	101	Forest, scrubland	Yes	[115,248]
Tapirus indicus	426250	Forest, grassland	Yes	[249,250]
Tayassu pecari	32500	Forest, grassland, scrubland, savanna	No	[115,251]
Theropithecus gelada	17050	Grassland, rocky areas	No	[115,252]
Thylamys elegans	50	Scrubland, forest	No	[115,253]
Tremarctos ornatus	108375	Forest, scrubland, grassland,	Yes	[26,254]
Tupaia glis	120	Forest	Yes	[125,255]
Ursus arctos	204500	Forest, wetlands, grassland, scrubland	No	[115,256]
Ursus maritimus	352500	Scrubland, forest, grassland, marine Intertidal	No	[115,257]
Varecia rubra	3450	Forest	Yes	[115,258]
Varecia variegata	3575	Forest	Yes	[115,259]
Vicugna pacos	63	Grassland, wetlands, scrubland, desert	No	[123,260]
Vicugna vicugna	50000^	Grassland, wetlands, scrubland, desert	No	[42,260]
Vulpes corsac	2325	Desert, grassland, scrubland	No	[114,261]

Vulpes lagopus	3200	Grassland	No	[114,262]
Vulpes vulpes	4835	Scrubland, grassland, wetlands, forest, desert	No	[125,263]

^ Mass calculated as the mid point between reported minimum and maximum values.

Supplementary Table 4. Model output for a phylogenetic generalized linear mixed model examining peak hearing sensitivity versus habitat with log_{10} head size as a covariate. Three separate MCMC chains were run. Model averaged values (in bold) are reported in the results section of the main text.

Chain	DIC	H^2 [95%CI]	Random (phylogeny)		Intercept (forest mammals)			Other mammals			Log_{10} head size			SRF
			ESS	Estimate [95%CI]	ESS	Estimate [95%CI]	P_{MCMC}	ESS	Estimate [95%CI]	P_{MCMC}	ESS	Estimate [95%CI]	P_{MCMC}	
1	304.25	0.09 [0.00, 0.43]	1090	2.74 [0.00, 12.90]	1090	13.56 [3.68, 23.80]	0.011	1090	-3.24 [-6.27, -0.56]	0.035	1090	-1.50 [-5.42, 2.70]	0.448	-
2	304.25	0.09 [0.00, 0.47]	1090	2.77 [0.00, 13.78]	1357	13.50 [2.67, 24.11]	0.018	1090	-3.18 [-5.88, -0.47]	0.026	1090	-1.50 [-5.86, 2.67]	0.473	-
3	304.25	0.09 [0.00, 0.44]	1090	2.52 [0.00, 12.78]	1090	13.30 [3.68, 22.78]	0.017	1090	-3.19 [-5.74, -0.38]	0.028	1090	-1.42 [-5.36, 2.22]	0.468	-
**Average**	**304.25**	**0.09 [0.00, 0.45]**	**1090**	**2.68 [0.00, 13.15]**	**1179**	**13.45 [3.43, 23.56]**	**0.015**	**1090**	**-3.20 [-5.96, -0.47]**	**0.030**	**1090**	**-1.47 [-5.55, 2.53]**	**0.463**	**1.00**

DIC, deviance information criteria; ESS, effective sample size; H^2, phylogenetic heritability; P_{MCMC}, likelihood that posterior probability distributions cross zero; SRF, scale reduction factor from a Gelman-Rubin test.

Supplementary Table 5. Model output for a phylogenetic generalized linear mixed model examining relative high frequency hearing sensitivity versus habitat with log_{10} head size as a covariate. Three separate MCMC chains were run. Model averaged values (in bold) are reported in the results section of the main text.

Chain	DIC	H^2 [95%CI]	Random (phylogeny)		Intercept (forest mammals)			Other mammals			Log_{10} head size			SRF
			ESS	Estimate [95%CI]	ESS	Estimate [95%CI]	P_{MCMC}	ESS	Estimate [95%CI]	P_{MCMC}	ESS	Estimate [95%CI]	P_{MCMC}	
1	316.85	0.76 [0.34, 1.00]	936	85.39 [0.00, 160.00]	1090	57.07 [36.00, 77.80]	<0.001	1090	-5.85 [-10.36, -1.09]	0.018	992	-19.03 [-26.84, -10.66]	<0.001	
2	316.02	0.75 [0.34, 1.00]	1090	83.89 [0.00, 155.80]	898	56.77 [34.20, 78.40]	<0.001	1417	-5.79 [-10.69, -1.41]	0.024	1090	-18.96 [-26.75 -11.33]	<0.001	
3	316.50	0.74 [0.31, 1.00]	1090	82.54 [0.00, 157.70]	1090	56.56 [34.35, 76.20]	<0.001	1090	-5.81 [-10.85, -1.00]	0.020	914	-18.92 [-27.58, -10.94]	0.002	
**Average**	**316.46**	**0.75 [0.33, 1.00]**	**1039**	**83.94 [0.00, 157.83]**	**1026**	**56.80 [34.85, 77.47]**	**<0.001**	**1199**	**-5.82 [-10.63, -1.17]**	**0.021**	**999**	**-18.97 [-27.06, -10.98]**	**<0.001**	**1.00**

DIC, deviance information criteria; ESS, effective sample size; H^2, phylogenetic heritability; P_{MCMC}, likelihood that posterior probability distributions cross zero; SRF, scale reduction factor from a Gelman-Rubin test.

Supplementary Table 6. Model output for a phylogenetic generalized linear mixed model examining spectral slope versus habitat with $\log_{10}$ body mass entered as a covariate and presumed call function as a random factor. Three separate MCMC chains were run. Model averaged values (in bold) are reported in the results section of the main text.

Chain	DIC	H^2 [95%CI]	Random (phylogeny)		Random (presumed call function)		Intercept (forest mammals)			Other mammals			Log_{10} body mass			SRF
			ESS	Estimate [95%CI]	ESS	Estimate [95%CI]	ESS	Estimate [95%CI]	P_{MCMC}	ESS	Estimate [95%CI]	P_{MCMC}	ESS	Estimate [95%CI]	P_{MCMC}	
1	-228.69	0.18 [0.03, 0.39]	1090	0.00 [0.00, 0.00]	828	0.00 [0.00, 0.00]	1090	-0.43 [-0.53, -0.33]	<0.001	1090	-0.06 [-0.11, -0.02]	0.004	1090	0.00 [-0.02, 0.03]	0.857	-
2	-228.69	0.18 [0.03, 0.42]	1090	0.00 [0.00, 0.00]	1090	0.00 [0.00, 0.00]	1090	-0.42 [-0.53, -0.33]	<0.001	1090	-0.07 [-0.11, -0.03]	<0.001	1090	0.00 [-0.02, 0.03]	0.826	-
3	-228.69	0.18 [0.03, 0.40]	1266	0.00 [0.00, 0.00]	1216	0.00 [0.00, 0.00]	1090	-0.43 [-0.54, -0.34]	<0.001	1090	-0.07 [-0.11, -0.03]	0.006	1090	0.00 [-0.02, 0.02]	0.817	-
**Average**	**-228.69**	**0.18 [0.03, 0.40]**	**1149**	**0.00 [0.00, 0.00]**	**1045**	**0.00 [0.00, 0.00]**	**1090**	**-0.43 [-0.54, -0.33]**	**<0.001**	**1090**	**-0.07 [-0.11, -0.02]**	**0.003**	**1090**	**0.00 [-0.02, 0.03]**	**0.833**	**1.00**

DIC, deviance information criteria; ESS, effective sample size; H^2, phylogenetic heritability; P_{MCMC}, likelihood that posterior probability distributions cross zero; SRF, scale reduction factor from a Gelman-Rubin test.

Supplementary Table 7. Model output for a phylogenetic generalized linear mixed model examining the relationship between spectral slope and peak hearing sensitivity. Three separate MCMC chains were run. Model averaged values (in bold) are reported in the results section of the main text.

Chain	DIC	H^2 [95%CI]	Random (phylogeny)		Intercept			Peak hearing sensitivity			SRF
			ESS	Estimate [95%CI]	ESS	Estimate [95%CI]	P_{MCMC}	ESS	Estimate [95%CI]	P_{MCMC}	
1	-26.38	0.33 [0.02, 0.80]	1090	0.00 [0.00, 0.02]	1090	-0.28 [-0.43, -0.11]	0.006	1090	-0.02 [-0.03, -0.00]	0.015	-
2	-26.40	0.34 [0.02, 0.81]	1090	0.00 [0.00, 0.02]	987	-0.27 [-0.43, -0.11]	0.013	983	-0.02 [-0.03, -0.00]	0.009	-
3	-26.41	0.32 [0.01, 0.76]	1090	0.00 [0.00, 0.02]	1090	-0.28 [-0.45, -0.12]	0.002	1090	-0.02 [-0.03, -0.00]	0.013	-
**Average**	**-26.40**	**0.33 [0.02, 0.79]**	**1090**	**0.00 [0.00, 0.02]**	**1056**	**-0.27 [-0.43, -0.11]**	**0.007**	**1054**	**-0.02 [-0.03, -0.00]**	**0.012**	**1.00**

DIC, deviance information criteria; ESS, effective sample size; H^2, phylogenetic heritability; P_{MCMC}, likelihood that posterior probability distributions cross zero; SRF, scale reduction factor from a Gelman-Rubin test.

Supplementary Table 8. Model output for a phylogenetic generalized linear mixed model examining the relationship between spectral slope and high frequency hearing sensitivity. Three separate MCMC chains were run. Model averaged values (in bold) are reported in the results section of the main text.

Chain	DIC	H^2 [95%CI]	Random (species)		Intercept			High frequency hearing sensitivity			SRF
			ESS	Estimate [95%CI]	ESS	Estimate [95%CI]	P_{MCMC}	ESS	Estimate [95%CI]	P_{MCMC}	
1	-23.23	0.24 [0.01, 0.62]	1090	0.00 [0.00, 0.02]	1090	-0.36 [-0.48, -0.24]	<0.001	1090	-0.01 [-0.02, -0.00]	0.049	-
2	-23.22	0.24 [0.01, 0.62]	1090	0.00 [0.00, 0.02]	1090	-0.36 [-0.49, -0.24]	<0.001	1090	-0.01 [-0.02, -0.00]	0.044	-
3	-23.23	0.23 [0.01, 0.61]	1090	0.00 [0.00, 0.02]	1090	-0.36 [-0.48, -0.23]	<0.001	1090	-0.01 [-0.02, -0.00]	0.040	-
**Average**	**-23.23**	**0.24 [0.01, 0.61]**	**1090**	**0.00 [0.00, 0.02]**	**1090**	**-0.36 [-0.48, -0.24]**	**<0.001**	**1090**	**-0.01 [-0.02, -0.00]**	**0.044**	**1.00**

DIC, deviance information criteria; ESS, effective sample size; H^2, phylogenetic heritability; P_{MCMC}, likelihood that posterior probability distributions cross zero; SRF, scale reduction factor from a Gelman-Rubin test.

Supplementary References

1 Bininda-Emonds, O. R. P. *et al*. The delayed rise of present-day mammals. *Nature* **446**, 507-512 (2007).

2 Heffner, R. S., Koay, G. & Heffner, H. E. Audiograms of five species of rodents: implications for the evolution of hearing and the perception of pitch. *Hear. Res.* **157**, 138-152 (2001).

3 Owen, M. A. *et al*. Hearing sensitivity in context: conservation implications for a highly vocal endangered species. *Glob. Ecol. Conserv.* **6**, 121-131 (2016).

4 Beecher, M. D. Hearing in the owl monkey (*Aotus trivirgatus*): I. Auditory sensitivity. *J. Comp. Physiol. Psychol.* **86**, 898-901 (1974).

5 Osmanski, M. S. & Wang, X. Measurement of absolute auditory thresholds in the common marmoset (*Callithrix jacchus*). *Hear. Res.* **277**, 127-133 (2011).

6 Ramsier, M. A., Vinyard, C. J. & Dominy, N. J. Auditory sensitivity of the tufted capuchin (*Sapajus apella*), a test of allometric predictions. *J. Acoust. Soc. Am.* **141**, 4822-4831 (2017).

7 Brown, C. H. & Waser, P. M. Hearing and communication in blue monkeys (*Cercopithecus mitis*). *Anim. Behav.* **32**, 66-75 (1984).

8 Coleman, M. N. *The functional morphology and evolution of the primate auditory system* PhD thesis, State University of New York at Stony Brook, (2007).

9 Owren, M. J., Hopp, S. L., Sinnott, J. M. & Petersen, M. R. Absolute auditory thresholds in three Old World monkey species (*Cercopithecus aethiops, C. neglectus, Macaca fuscata*) and humans (*Homo sapiens*). *J. Comp. Psychol.* **102**, 99-107 (1988).

10 Heffner, R. S. & Heffner, H. E. Behavioral hearing range of the chinchilla. *Hear. Res.* **52**, 13-16 (1991).

11 Heffner, R. S., Heffner, H. E., Contos, C. & Kearns, D. Hearing in prairie dogs: transition between surface and subterranean rodents. *Hear. Res.* **73**, 185-189 (1994).

12 Ravizza, R. J., Heffner, H. E. & Masterton, B. Hearing in primitive mammals: I. Opossum (*Didelphis virginianus*). *J. Aud. Res.* **9**, 1-7 (1969).

13 Ravizza, R. J. & Masterton, B. Contribution of neocortex to sound localization in opossum (*Didelphis virginiana*). *J. Neurophysiol.* **35**, 344-356 (1972).

14 Webster, D. B. & Webster, M. Kangaroo rat auditory thresholds before and after middle ear reduction. *Brain Behav. Evol.* **5**, 41-53 (1972).

15 Heffner, H. & Masterton, B. Hearing in Glires: domestic rabbit, cotton rat, feral house mouse, and kangaroo rat. *J. Acoust. Soc. Am.* **68**, 1584-1599 (1980).

16 Heffner, R. S. & Heffner, H. E. Hearing in the elephant (*Elephas maximus*): absolute sensitivity, frequency discrimination, and sound localization. *J. Comp. Physiol. Psychol.* **96**, 926-944 (1982).

17 Ramsier, M. A., Cunningham, A. J., Finneran, J. J. & Dominy, N. J. Social drive and the evolution of primate hearing. *Philos. Trans. R. Soc. Lond. Ser. B-Biol. Sci.* **367**, 1860-1868 (2012).

18 Heffner, H. E., Ravizza, R. J. & Masterton, B. Hearing in primitive mammals: IV. Bushbaby (*Galago senegalensis*). *J. Aud. Res.* **9**, 19-23 (1969).

19 Ravizza, R. J., Heffner, H. E. & Masterton, B. Hearing in primitive mammals: II. Hedgehog (*Hemiechinus auritus*). *J. Aud. Res.* **9**, 8-11 (1969).

20 Gillette, R. G., Brown, R., Herman, P., Vernon, S. & Vernon, J. The auditory sensitivity of the lemur. *Am. J. Phys. Anthropol.* **38**, 365-370 (1973).

21 Fujita, S. & Elliott, D. N. Thresholds of audition for three species of monkeys. *J. Acoust. Soc. Am.* **36**, 1007-1007 (1964).

22 Coleman, M. N. What do primates hear? A meta-analysis of all known nonhuman primate behavioral audiograms. *Int. J. Primatol.* **30**, 55-91 (2009).

23 Jackson, L. L., Heffner, R. S. & Heffner, H. E. Free-field audiogram of the Japanese macaque (*Macaca fuscata*). *J. Acoust. Soc. Am.* **106**, 3017-3023 (1999).

24 Stebbins, W. C., Green, S. & Miller, F. L. Auditory sensitivity of the monkey. *Science* **153**, 1646-1647 (1966).

25 Ryan, A. Hearing sensitivity of the mongolian gerbil, *Meriones unguiculatis*. *J. Acoust. Soc. Am.* **59**, 1222-1226 (1976).

26 Frost, S. B. & Masterton, R. B. Hearing in primitive mammals: *Monodelphis domestica* and *Marmosa elegans*. *Hear. Res.* **76**, 67-72 (1994).

27 Heffner, R. S. & Heffner, H. E. Hearing in mammals: the least weasel. *J. Mammal.* **66**, 745-755 (1985).

28 Kelly, J. B., Kavanagh, G. L. & Dalton, J. C. H. Hearing in the ferret (*Mustela putorius*): thresholds for pure tone detection. *Hear. Res.* **24**, 269-275 (1986).

29 Heffner, H. E. & Heffner, R. S. Hearing in two cricetid rodents: wood rat (*Neotoma floridana*) and grasshopper mouse (*Onychomys leucogaster*). *J. Comp. Psychol.* **99**, 275-288 (1985).

30 Heffner, H. & Masterton, B. Hearing in primitive primates: Slow loris (*Nycticebus coucang*) and potto (*Perodicticus potto*). *J. Comp. Physiol. Psychol.* **71**, 175-182 (1970).

31 Heffner, H. & Heffner, H. E. The behavioral audiogram of whitetail deer (*Odocoileus virginianus*). *J. Acoust. Soc. Am.* **127**, EL111-EL114, (2010).

32 Kojima, S. Comparison of auditory functions in the chimpanzee and human. *Folia Primatol.* **55**, 62-72, doi:10.1159/000156501 (1990).

33 Hienz, R. D., Turkkan, J. S. & Harris, A. H. Pure tone thresholds in the yellow baboon (*Papio cynocephalus*). *Hear. Res.* **8**, 71-75 (1982).

34 Wollack, C. H. Auditory thresholds in the raccoon (*Procyon lotor*). *J. Aud. Res.* **5**, 139-144 (1965).

35 Flydal, K., Hermansen, A., Enger, P. S. & Reimers, E. Hearing in reindeer (Rangifer tarandus). *J. Comp. Physiol. A-Sens. Neural Behav. Physiol.* **187**, 265-269 (2001).

36 Heffner, H. E., Heffner, R. S., Contos, C. & Ott, T. Audiogram of the hooded Norway rat. *Hear. Res.* **73**, 244-247 (1994).

37 Beecher, M. D. Pure-tone thresholds of the squirrel monkey (*Saimiri sciureus*). *J. Acoust. Soc. Am.* **55**, 196-198 (1974).

38 Green, S. Auditory sensitivity and equal loudness in the squirrel monkey (*Saimiri sciureus*). *J. Exp. Anal. Behav.* **23**, 255-264 (1975).

39 Heffner, R. S. & Heffner, H. E. Hearing in domestic pigs (*Sus scrofa*) and goats (*Capra hircus*). *Hear. Res.* **48**, 231-240 (1990).

40 Heffner, H. E., Ravizza, R. J. & Masterton, B. Hearing in primitive mammals: III. Tree shrew (*Tupaia glis*). *J. Aud. Res.* **9**, 12-18 (1969).

41 Owen, M. & Bowles, A. E. In-air auditory psychophysics and the management of a threatened carnivore, the polar bear (*Ursus maritimus*). *Int. J. Comp. Psychol.* **24**, 244-254 (2011).

42 Heffner, R. S., Koay, G. & Heffner, H. E. Hearing in alpacas (*Vicugna pacos*): audiogram, localization acuity, and use of binaural locus cues. *J. Acoust. Soc. Am.* **135**, 778-788 (2014).

43 Malkemper, E. P., Topinka, V. & Burda, H. A behavioral audiogram of the red fox (*Vulpes vulpes*). *Hear. Res.* **320**, 30-37 (2015).

44 Smirnova, D. S., Volodin, I. A., Demina, T. S. & Volodina, E. V. Acoustic structure and contextual use of calls by captive male and female cheetahs (*Acinonyx jubatus*). *PLoS ONE* **11**, e0158546-0158520, doi:10.1371/journal.pone.0158546 (2016).

45 Kleiman, D. G. & Peters, G. in *Second International Symposium on the Giant Panda.* (eds S. Asakura & S Nakagawa) 107-122 (Tokyo Zoological Park Society).

46 Cap, H., Deleporte, P., Joachim, J. & Reby, D. Male vocal behavior and phylogeny in deer. *Cladistics* **24**, 917-931 (2008).

47 Kumar, S. & Rahmani, A. R. Predation by wolves (*Canis lupus pallipes*) on blackbuck (*Antilope cervicapra*) in the great Indian Bustard Sanctuary, Nannaj, Maharashtra, India. *Int. J. Ecol. Env. Sci.* **34**, 99-112 (2008).

48 Puts, D. A. *et al.* Sexual selection on male vocal fundamental frequency in humans and other anthropoids. *Proc. R. Soc. B.* **283**, 20152830 (2016).

49 Berger, J. & Cunningham, C. Bellows, copulations, and sexual selection in bison (*Bison bison*). *Behav. Ecol.* **2**, 1 (1991).

50 Sankar, K., Johnsingh, A. J. T. & Acharya, B. in *Ungulates of India* Vol. 7 (eds K. Sankar & S. P. Goyal) (Wildlife Institue of India, 2004).

51 Frey, R. & Hofmann, R. R. Larynx and vocalization of the takin (*Budorcas taxicolor* Hodgson, 1850-Mammalia, Bovidae). *Z. Anz.* **239**, 197-214 (2000).

52 De La Torre, S. & Snowdon, C. T. Dialects in pygmy marmosets? Population variation in call structure. *Am. J. Primatol.* **71**, 333-342 (2009).

53 Kiley, M. The vocalisations of ungulates, their causation and function. *Z. Tierpsychol.* **31**, 171-222 (1972).

54 Tembrock, G. Canid vocalizations. *Behav. Processes* **1**, 57-75 (1976).

55 Mitchell, B. R., Makagon, M. M., Jaeger, M. M. & Barrett, R. H. Information content of coyote barks and howls. *Bioacoustics* **15**, 289-314 (2006).

56 Reby, D. & Cargnelutti, B. Des voix dans la foreˆt. Un guide sonore des Cervides d'Europe. Sittelle, Mens (1999).

57 Gros-Louis, J. J. *et al*. Vocal repertoire of *Cebus capucinus*: acoustic structure, context, and usage. *Int. J. Primatol.* **29**, 641-670 (2008).

58 Zuberbühler, K., Noë, R. & Seyfarth, R. Diana monkey long-distance calls: messages for conspecifics and predators. *Anim. Behav.* **53**, 589-604 (1997).

59 Fuller, J. L. The vocal repertoire of adult male blue monkeys (*Cercopithecus mitis stulmanni*): A quantitative analysis of acoustic structure. *Am. J. Primatol.* **76**, 203-216 (2014).

60 Bouchet, H., Blois-Heulin, C. & Lemasson, A. Age- and sex-specific patterns of vocal behavior in De Brazza's monkeys (*Cercopithecus neglectus*). *Am. J. Primatol.* **74**, 12-28 (2012).

61 Brady, C. A. The vocal repertoires of the bush dog (*Speothos venaticus*), crab-eating fox (*Cerdocyon thous*), and maned wolf (*Chrysocyon brachyurus*). *Anim. Behav.* **29**, 649-669 (1981).

62 Harris, T. R., Fitch, W. T., Goldstein, L. M. & Fashing, P. J. Black and white colobus monkey (*Colobus guereza*) roars as a source of both honest and exaggerated information about body mass. *Ethology* **112**, 911-920 (2006).

63 East, M. L. & Hofer, H. Loud Calling in a Female-Dominated Mammalian Society .2. Behavioral Contexts and Functions of Whooping of Spotted Hyaenas, Crocuta-Crocuta. *Anim. Behav.* **42**, 651-669 (1991).

64 Budde, C. & Klump, G. M. Vocal repetiore of the black rhino *Diceros bicornis* ssp. and possibilities of individual identification. *Mammalian Biology* **68**, 42-47 (2003).

65 De Silva, S. Acoustic communication in the Asian elephant, *Elephas maximus Behaviour* **147**, 825-852 (2010).

66 Alberghina, D., Caudullo, E., Bandi, N. & Panzera, M. A comparative analysis of the acoustic structure of separation calls of Mongolian wild horses (*Equus ferus przewalskii*) and domestic horses (*Equus caballus*). *Journal of Veterinary Behavior: Clinical Applications and Research* **9**, 254-257 (2014).

67 Churcher, C. S. Equus grevyi. *Mam. Sp.* **453**, 1-9 (1993).

68 Saunders, A. A. The Voice of the Porcupine. *J. Mammal.* **13**, 167-168 (1932).

69 Attie, C. Emissions sonores chez le Hérisson européen, *Erinaceus europaeus*, et signification comportementale. *Mammalia* **54**, 3-12 (2009).

70 Peters, G., Baum, L., Peters, M. & Tonkin-Leyhausen, B. Spectral characteristics of intense mew calls in cat species of the genus Felis (*Mammalia: Carnivora: Felidae)*. *J. Ethol.* **27**, 221-237 (2009).

71 Estes, R. *The Safari Companion*. (Green Publishing Company, 1993).

72 Pasitschniak-Arts, M. & Lariviere, S. Gulo gulo. *Mam. Sp.* **499**, 1-10 (1995).

73 Barelli, C., Mundry, R., Heistermann, M. & Hammerschmidt, K. Cues to androgens and quality in male gibbon songs. *PLoS ONE* **8**, e82748, doi: 10.1371/journal.pone.0082748 (2013).

74 Brockelman, W., Geissmann, T., Timmins, T. & Traeholt, C. *Hylobates pileatus*. *The IUCN Red List of Threatened Species*, e.T10552A3200582 (2008).

75 Casamitjana, J. The vocal repertoire of the woolly monkey *Lagothrix lagothricha*. *Bioacoustics* **13**, 1-19 (2002).

76 Charlton, B. Vocal distinctiveness in the harsh coughs of southern hairy-nosed wombats (*Lasiorhinus latifrons*). *Acta Acustica United With Acustica* **100**, 719-723 (2014).

77 Macedonia, J. The vocal repertoire of the ringtailed lemur (*Lemur catta*). *Folia Primatol.* **61**, 186-217 (1993).

78 McLanahan, E. B. & Green, K. N. in *The biology and conservation of the Callitrichidae* (ed D. Kleiman) 251-269 (Smithsonian Institution Press, 1977).

79 Sunquist, M. & Sunquist, F. *Wild cats of the world*. (University of Chicago Press, 2002).

80 Soltis, J. Vocal communication in African Elephants (*Loxodonta africana*). *Zoo Biology*, **29**, 192-209 (2009).

81 Peters, G. Acoustic communication in the genus Lynx (Mammalia: Felidae)—comparative survey and phylogenetic interpretation. *Bonn. zool. Beitr* **38**, 315-330 (1987).

82 Palombit, R. A. A preliminary study of vocal communication in wild long-tailed macaques (*Macaca fascicularis*). II. Potential of calls to regulate intragroup spacing. *Int. J. Primatol.* **13**, 183-207 (1992).

83 Green, S. in *Primate behavior* Vol. 4 (ed L. A. Rosenblum) (Academic Press, 1975).

84 Grimm, R. J. Catalogue of sounds of the Pigtailed macaque (*Macaca nemestrina*). *J. Zool.* **152**, 361-373 (1967).

85 Hohmann, G. M. & Herzog, M. O. Vocal communication in lion-tailed macaques (*Macaca silenus*). *Folia Primatol.* **45**, 148-178 (1985).

86 Kudo, H. The study of vocal communication of wild mandrills in Cameroon in relation to their social structure. *Primates* **28**, 289-308 (1987).

87 Laurie, A. & Seidensticker, J. Behavioural ecology of the Sloth bear (*Melursus ursinus*). *J. Zool.* **182**, 187-204 (1977).

88 Furrer, R. D. & Manser, M. B. Banded mongoose recruitment calls convey information about risk and not stimulus type. *Anim. Behav.* **78**, 195-201 (2009).

89 Woods, C. A., Contreras, L., Willner-Chapman, G. & Whidden, H. P. Myocastor coypus. *Mam. Sp.*, 1-8 (1992).

90 Gasco, A., Ferro, H. F. & Monticelli, P. F. The communicative life of a social carnivore: acoustic repertoire of the ring-tailed coati (*Nasua nasua*). *Bioacoustics*, 1-29 (2018).

91 Fan, P. F., Xiao W Fau - Huo, S., Huo S Fau - Jiang, X.-L. & Jiang, X. L. Singing behavior and singing functions of black-crested gibbons (*Nomascus concolor jingdongensis*) at Mt. Wuliang, central Yunnan, China. *Am. J. Primatol.* **71**, 539-547.

92 Long, C. V. Vocalisations of the Degu *Octodon degus*, a social caviomorph rodent. *Bioacoustics* **16**, 223-244 (2007).

93 Frey, R., Gebler, A. & Fritsch, G. Arctic roars - laryngeal anatomy and vocalization of the muskox (*Ovibos moschatus* Zimmermann, 1780, *Bovidae*). *J. Zool.* **268**, 433-448 (2006).

94 Bermejo, M. & Omedes, A. Preliminary Vocal Repertoire and Vocal Communication of Wild Bonobos (*Pan paniscus*) at Lilungu (Democratic Republic of Congo). *Folia Primatol*. **70**, 328-357 (1999).

95 Goodall, J. *The chimpanzees of Gombe*. (Belknapp, 1986).

96 Estes, R. D. *The behavior guide to African mammals*. (University of California Press, 1991).

97 Byers, J. A. & Bekoff, M. Social, spacing, and cooperative behavior of the collared peccary, *Tayassu tajacu*. *J. Mammal*. **62**, 767-785 (1981).

98 Smith, M. Behaviour of the koala, *Phascolarctos cinereus* (Goldfuss), in captivity. III. Vocalizations. *Aust. Wild. Res*. **7**, 13-34 (1980).

99 Hardus, M. *et al*. in *Orangutans: Geographic Variation in Behavioral Ecology and Conservation* (eds Wich, S. A., Atmoko, S. S. U., Setia, T. M., van Schaik, C. P.) Ch. 4, (Oxford University Press, 2008).

100 Huffman, B. A. & Leslie, D. M., Jr. Potamochoerus porcus (Artiodactyla: Suidae). *Mam. Sp*. **47**, 15-31 (2015).

101 Poglayen-Neuwall, I. Beiträge zu einem Ethogramm des Wickelbären (*Potos flavus Schreber*). *Z. Säugetierkd*. **21**, 1-64 (1962).

102 Sieber, O. J. Vocal communication in raccoons (*Procyon lotor*). *Behaviour* **90**, 80-113 (1984).

103 Cleveland, J. & Snowdon, C. T. The complex vocal repertoire of the adult cotton-top tamarin (*Saguinus oedipus oedipus*). *Z. Tierpsychol*. **58**, 231-270 (1982).

104 Winter, P., Ploog, D. & Latta, J. Vocal repertoire of the squirrel monkey (*Saimiri sciureus*), its analysis and significance. *Exp Brain Res* **1**, 359-384 (1966).

105 Bhaker, N. R., Rajpurohit, D. S. & Rajpurohit, L. S. Vocalization in Hanuman langur, *Semnopithecus entellus* around Jodhpur, Rajasthan. *Uttar Pradesh Journal of Zoology* **24**, 227-233 (2004).

106 Manser, M. B. The acoustic structure of suricates' alarm calls varies with predator type and the level of response urgency. *Proc. R. Soc. B*. **268**, 2315-2324 (2001).

107 Cowlishaw, G. Song function in gibbons. *Behaviour* **121**, 131-153 (1992).

108 Cornélis, D. *et al*. in *Ecology, Evolution and Behaviour of Wild Cattle: Implications for Conservation* (eds James Burton & Mario Melletti) 326-372 (Cambridge University Press, 2014).

109 Lissovsky, A. A., Obolenskaya, E. V. & Emelyanova, L. G. The structure of voice signals of Siberian chipmunk (*Tamias sibiricus* Laxmann 1769; Rodentia: Sciuridae). *Russ. J. Theriol.* **5**, 93-98 (2006).

110 Gustison, M. L., le Roux, A. & Bergman, T. J. Derived vocalizations of geladas (*Theropithecus gelada*) and the evolution of vocal complexity in primates. *Philos. Trans. R. Soc. Lon. B.* **367**, 1847-1859 (2012).

111 Esser, D., Zimmermann, E. & Schehka, S. Species-specificity in communication calls of tree shrews (*Tupaia*: Scandentia). *J. Mammal.* **89**, 1456-1463 (2008).

112 Jonkel, C. The behavior of captured North American bears (with comments on bear management and research). *BioScience* **20**, 1145-1147 (1970).

113 Pereira, M. E., Seeligson, M. L. & Macedonia, J. The behavioral repertoire of the black-and-white ruffed lemur, *Varecia variegata variegata* (Primates: Lemuridae). *Folia Primatol.* **51**, 1-32 (1988).

114 Clark, H. O., Murdoch Jr, J. D., Newman, D. P. & Sillero-Zubiri, C. *Vulpes corsac* (Carnivora: Canidae). *Mamm. Sp.* **832**, 1-8 (2009).

115 Silva, M. & Downing, J. A. *CRC handbook of mammalian body masses*. (CRC press, 1995).

116 Durant, S., Mitchell, N., Ipavec, A. & Groom, R. *Acinonyx jubatus. The IUCN Red List of Threatened Species* (2015).

117 Cassola, F. *Acomys cahirinus* (errata version published in 2017). *The IUCN Red List of Threatened Species*, e.T263A115048396 (2016).

118 Group, I. S. A. S. *Addax nasomaculatus. The IUCN Red List of Threatened Species,* e.T512A50180603 (2016).

119 Iossa, G., Soulsbury, C. D., Baker, P. J. & Harris, S. Sperm competition and the evolution of testes size in terrestrial mammalian carnivores. *Funct Ecology* **22**, 655-662 (2008).

120 Swaisgood, R., Wang, D. & Wei, F. *Ailuropoda melanoleuca. The IUCN Red List of Threatened Species,* e.T712A121745669 (2016).

121 Hundertmark, K. *Alces alces. The IUCN Red List of Threatened Species*, e.T56003281A22157381 (2016).

122 Group, I. S. A. S. *Antilope cervicapra. The IUCN Red List of Threatened Species,* e.T1681A50181949 (2017).

123 Smith, R. J. & Junkers, W. L. Body mass in comparative primatology. *J. Hum. Evol.* **32**, 523-559 (1997).

124 Urbani, B., Lynch Alfaro, J. & de Azevedo , R. *Aotus trivirgatus*. *The IUCN Red List of Threatened Species* e.T41543A17923788 (2018).

125 Jones, K. E. et al. PanTHERIA: a species-level database of life history, ecology, and geography of extant and recently extinct mammals. *Ecology* **90** 2648-2648 (2009).

126 Willcox, D. H. A. *et al*. *Arctictis binturong*. *The IUCN Red List of Threatened Species* e.T41690A45217088 (2016).

127 Cuarón, A. D. *et al*. *Ateles geoffroyi*. *The IUCN Red List of Threatened Species,* e.T2279A9387270 (2008).

128 Aune, K., Jørgensen, D. & Gates, C. *Bison bison* (errata version published in 2018). *The IUCN Red List of Threatened Species*, e.T2815A123789863 (2017).

129 Group, I. S. A. S. *Boselaphus tragocamelus*. *The IUCN Red List of Threatened Species,* e.T2893A115064758 (2016).

130 Song, Y. L., Smith, A. T. & MacKinnon, J. *Budorcas taxicolor*. *The IUCN Red List of Threatened Species* e.T3160A9643719 (2008).

131 Bezerra, B. *et al*. *Callithrix jacchus*. *The IUCN Red List of Threatened Species*, e.T41518A17936001 (2018).

132 de la Torre, S. & Rylands, A. B. *Callithrix pygmaea*. *The IUCN Red List of Threatened Species,* e.T41535A10493764 (2008).

133 Hare, J. *Camelus ferus*. *The IUCN Red List of Threatened Species* (2008).

134 Hoffmann, M. *et al*. *Canis aureus*. *The IUCN Red List of Threatened Species,* e.T118264161A46194820 (2018).

135 Kays, R. *Canis latrans*. *The IUCN Red List of Threatened Species*, e.T3745A103893556 (2018).

136 Boitani, L., Phillips, M. & Jhala, Y. *Canis lupus*. *The IUCN Red List of Threatened Species*, e.T3746A119623865 (2018).

137 Lovari, S. *et al*. *Capreolus capreolus*. *The IUCN Red List of Threatened Species*, e.T42395A22161386 (2016).

138 Boubli, J. *et al*. *Sapajus apella*. *The IUCN Red List of Threatened Species*, e.T39949A70611337 (2018).

139 Oates, J. F., Gippoliti, S. & Groves, C. P. *Cercopithecus diana*. *The IUCN Red List of Threatened Species* e.T4245A92376689 (2016).

140 Kingdon, J. *et al*. *Cercopithecus mitis*. *The IUCN Red List of Threatened Species*, e.T4221A10676022 (2008).

141 Struhsaker, T., Oates, J. F., Hart, J. & Butynski, T. M. *Cercopithecus neglectus*. *The IUCN Red List of Threatened Species* e.T4223A10680717 (2008).

142 Lovari, S. *et al*. *Cervus elaphus*. *The IUCN Red List of Threatened Species*, e.T55997072A142404453 (2018).

143 Harris, R. B. *Cervus nippon*. *The IUCN Red List of Threatened Species,* e.T41788A22155877 (2015).

144 Spotorno, A. E., Zuleta, C. A., Valladares, J. P., Deane, A. L. & Jiménez, J. E. Chinchilla laniger. *Mam. Sp*., **758**, 1-9 (2004).

145 Roach, N. & Kennerley, R. *Chinchilla lanigera*. *The IUCN Red List of Threatened Species* e.T4652A117975205 (2016).

146 Kingdon, J. & Butynski, T. M. *Chlorocebus aethiops*. *The IUCN Red List of Threatened Species*, e.T4233A10695029 (2008).

147 Paula, R. C. & DeMatteo, K. *Chrysocyon brachyurus*. *The IUCN Red List of Threatened Species* e.T4819A88135664 (2015).

148 Kingdon, J., Struhsaker, T., Oates, J. F., Hart, J. & Groves, C. P. *Colobus guereza*. *The IUCN Red List of Threatened Species*, e.T5143A11116447 (2008).

149 Vrahimis, S., Grobler, P., Brink, J., Viljoen, P. & Schulze, E. *Connochaetes gnou*. *The IUCN Red List of Threatened Species* e.T5228A50184962 (2017).

150 Group, I. S. A. S. *Connochaetes taurinus*. *The IUCN Red List of Threatened Species,* e.T5229A50185086 (2016).

151 Bohm, T. & Höner, O. R. *Crocuta crocuta*. *The IUCN Red List of Threatened Species*, e.T5674A45194782 (2015).

152 Cassola, F. *Cynomys leucurus*. *The IUCN Red List of Threatened Species*, e.T42454A22261371 (2016).

153 Cassola, F. *Cynomys ludovicianus*. *The IUCN Red List of Threatened Species*, e.T6091A115080297 (2016).

154 Masseti, M. & Mertzanidou, D. *Dama dama*. *The IUCN Red List of Threatened Species*, e.T42188A10656554 (2008).

155 Emslie, R. *Diceros bicornis*. *The IUCN Red List of Threatened Species,* e.T6557A16980917 (2012).

156 Pérez-Hernandez, R., Lew, D. & Solari, S. *Didelphis virginiana*. *The IUCN Red List of Threatened Species* e.T40502A22176259 (2016).

157 Timm, R., Álvarez-Castañeda, S. T. & Lacher, T. *Dipodomys merriami*. *The IUCN Red List of Threatened Species* e.T92465716A115515430 (2016).

158 Choudhury, A. *et al*. *Elephas maximus*. *The IUCN Red List of Threatened Species,* e.T7140A12828813 (2008).

159 King, S. R. B. & Moehlman, P. D. *Equus quagga*. *The IUCN Red List of Threatened Species*, e.T41013A45172424 (2016).

160 King, S. R. B., Boyd, L., Zimmermann, W. & Kendall, B. E. Equus ferus ssp. przewalskii *The IUCN Red List of Threatened Species*, e.T41763A97204950 (2015).

161 Rubenstein, D., Low Mackey, B., Davidson, Z. D., Kebede, F. & King, S. R. B. *Equus grevyi*. *The IUCN Red List of Threatened Species* e.T7950A89624491 (2016).

162 Kaczensky, P., Lkhagvasuren, B., Pereladova, O., Hemami, M. & Bouskila, A. *Equus hemionus*. *The IUCN Red List of Threatened Species*, e.T7951A45171204 (2015).

163 Emmons, L. *Erethizon dorsatum*. *The IUCN Red List of Threatened Species,* e.T8004A22213161 (2016).

164 Amori, G. *Erinaceus europaeus*. *The IUCN Red List of Threatened Species*, e.T29650A2791303 (2016).

165 Andriaholinirina, N. *et al*. *Eulemur fulvus*. *The IUCN Red List of Threatened Species*, e.T8207A16117505 (2014).

166 Andriaholinirina, N. *et al*. *Eulemur macaco*. *The IUCN Red List of Threatened Species*, e.T8212A16117639 (2014).

167 Andriaholinirina, N. *et al*. *Eulemur mongoz*. *The IUCN Red List of Threatened Species*, e.T8202A16117799 (2014).

168 Gray, T. N. E. *et al*. *Felis chaus*. *The IUCN Red List of Threatened Species,* e.T8540A50651463 (2014).

169 Yamaguchi, N., Kitchener, A., Driscoll, C. & Nussberger, B. *Felis silvestris*. *The IUCN Red List of Threatened Species* e.T60354712A50652361 (2015).

170 Bearder, S., Butynski, T. M. & De Jong, Y. *Galago senegalensis*. *The IUCN Red List of Threatened Species*, e.T8789A12932627 (2008).

171 Group, I. S. A. S. *Nanger dama*. *The IUCN Red List of Threatened Species,* e.T8968A50186128 (2016).

172 Cassola, F. *Geomys bursarius*. *The IUCN Red List of Threatened Species*, e.T42588A115192675 (2016).

173 Abramov, A. V. *Gulo gulo*. *The IUCN Red List of Threatened Species*, e.T9561A45198537 (2016).

174 Scotson, L. *et al*. *Helarctos malayanus*. *The IUCN Red List of Threatened Species* e.T9760A123798233 (2017).

175 Stubbe, M. *et al*. *Hemiechinus auritus*. *The IUCN Red List of Threatened Species* e.T40607A115174672 (2016).

176 AbiSaid, M. & Dloniak, S. M. D. *Hyaena hyaena*. *The IUCN Red List of Threatened Species*, e.T10274A45195080 (2015).

177 Harris, R. B. & Duckworth, J. W. *Hydropotes inermis*. *The IUCN Red List of Threatened Species,* e.T10329A22163569 (2015).
178 Brockelman, W. & Geissmann, T. *Hylobates lar*. *The IUCN Red List of Threatened Species*, e.T10548A3199623 (2008).
179 Brockelman, W., Geissmann, T., Timmins, T. & Traeholt, C. *Hystrix indica*. *The IUCN Red List of Threatened Species*, e.T10751A115099509 (2008).
180 Palacios, E., Boubli, J.-P., Stevenson, P., Di Fiore, A. & de la Torre, S. *Lagothrix lagotricha*. *The IUCN Red List of Threatened Species* e.T11175A3259920 (2008).
181 M Clarke, A. *et al*. Live animal measurements, carcass composition and plasma hormone and metabolite concentrations in male progeny of sires differing in genetic merit for beef production. *Animal* **3**, 933-945 (2009).
182 Baldi, R. B. *et al*. *Lama guanicoe*. *The IUCN Red List of Threatened Species*, e.T11186A18540211 (2016).
183 Woinarski, J. & Burbidge, A. A. *Lasiorhinus latifrons*. *The IUCN Red List of Threatened Species* e.T40555A21959203 (2016).
184 Kierulff, M. C. M., Rylands, A. B. & de Oliveira, M. M. *Leontopithecus rosalia*. *The IUCN Red List of Threatened Species*, e.T11506A3287321 (2008).
185 Paviolo, A. *et al*. *Leopardus pardalis*. *The IUCN Red List of Threatened Species* e.T11509A97212355 (2015).
186 Thiel, C. *Leptailurus serval*. *The IUCN Red List of Threatened Species* e.T11638A50654625 (2015).
187 Blanc, J. *Loxodonta africana*. *The IUCN Red List of Threatened Species*, e.T12392A3339343 (2008).
188 Kelly, M., Morin, D. & Lopez-Gonzalez, C. A. *Lynx rufus*. *The IUCN Red List of Threatened Species*, e.T12521A50655874 (2016).
189 Ong, P. & Richardson, M. *Macaca fascicularis*. *The IUCN Red List of Threatened Species* e.T12551A3355536 (2008).
190 Watanabe, K. & Tokita, K. *Macaca fuscata*. *The IUCN Red List of Threatened Species* e.T12552A3355997 (2008).
191 Timmins, R. J., Richardson, M., Chhangani, A. & Yongcheng, L. *Macaca mulatta*. *The IUCN Red List of Threatened Species* e.T12554A3356486 (2008).
192 Richardson, M., Mittermeier, R. A., Rylands, A. B. & Konstant, B. *Macaca nemestrina*. *The IUCN Red List of Threatened Species* e.T12555A3356892 (2008).

193 Kumar, A., Singh, M. & Molur, S. *Macaca silenus*. *The IUCN Red List of Threatened Species*, e.T12559A3358033 (2008).

194 Oates, J. F. & Butynski, T. M. *Mandrillus sphinx*. *The IUCN Red List of Threatened Species* e.T12754A3377579 (2008).

195 Cassola, F. *Marmota monax*. *The IUCN Red List of Threatened Species*, e.T42458A115189992 (2016).

196 Herrero, J. *et al*. *Martes martes*. *The IUCN Red List of Threatened Species,* e.T12848A45199169 (2016).

197 Dharaiya, N., Bargali, H. S. & Sharp, T. *Melursus ursinus*. *The IUCN Red List of Threatened Species,* e.T13143A45033815 (2016).

198 Batsaikhan, N. & Tsytsulina, K. *Meriones unguiculatus*. *The IUCN Red List of Threatened Species*, e.T13171A115110851 (2016).

199 Yigit, N. & Kryštufek, B. *Mesocricetus auratus*. *The IUCN Red List of Threatened Species* e.T13219A3421173 (2008).

200 Flores, D. & de la Sancha, N. *Monodelphis domestica*. *The IUCN Red List of Threatened Species,* e.T40514A22171137 (2016).

201 Gilchrist, J. S. & Do Linh San, E. *Mungos mungo*. *The IUCN Red List of Threatened Species,* e.T41621A45208886 (2016).

202 McDonald, R. A. *et al*. *Mustela nivalis*. *The IUCN Red List of Threatened Species*, e.T70207409A45200499 (2016).

203 Skumatov, D. *et al*. *Mustela putorius*. *The IUCN Red List of Threatened Species* e.T41658A45214384 (2016).

204 Ojeda, R., Bidau, C. & Emmons, L. *Myocastor coypus*. *The IUCN Red List of Threatened Species* e.T14085A121734257 (2016).

205 Emmons, L. & Helgen, K. *Nasua nasua*. *The IUCN Red List of Threatened Species,* e.T41684A45216227 (2016).

206 Grassman, L. *et al*. *Neofelis nebulosa*. *The IUCN Red List of Threatened Species,* e.T14519A97215090 (2016).

207 Cassola, F. *Neotoma floridana*. *The IUCN Red List of Threatened Species*, e.T42650A115199202 (2016).

208 Bleisch, B., Geissmann, T., Timmins, R. J. & Xuelong, J. *Nomascus concolor*. *The IUCN Red List of Threatened Species*, e.T39775A10265349 (2008).

209 Nekaris, A. & Streicher, U. *Nycticebus coucang*. *The IUCN Red List of Threatened Species* e.T39759A10263403 (2008).

210 Roach, N. *Octodon degus*. *The IUCN Red List of Threatened Species* e.T15088A78321302 (2016).

211 Sanchez Rojas, G. & Gallina Tessaro, S. *Odocoileus hemionus*. *The IUCN Red List of Threatened Species* e.T42393A22162113 (2016).

212 Gallina, S. & Lopez Arevalo, H. *Odocoileus virginianus*. *The IUCN Red List of Threatened Species,* e.T42394A22162580 (2016).

213 Timm, R. *Onychomys leucogaster*. *The IUCN Red List of Threatened Species* e.T15338A115127288 (2016).

214 Smith, A. T. & Boyer, A. F. *Oryctolagus cuniculus*. *The IUCN Red List of Threatened Species* e.T41291A10415170 (2008).

215 Group, I. S. A. S. *Oryx dammah*. *The IUCN Red List of Threatened Species,* e.T15568A50191470 (2016).

216 Gunn, A. & Forchhammer, M. *Ovibos moschatus*. *The IUCN Red List of Threatened Species,* e.T29684A86066477 (2008).

217 Harris, R. B. & Reading, R. *Ovis ammon*. *The IUCN Red List of Threatened Species,* e.T15733A5074694 (2008).

218 Fruth, B. *et al*. *Pan paniscus*. *The IUCN Red List of Threatened Species,* e.T15932A102331567 (2016).

219 Humle, T., Maisels, F., Oates, J. F., Plumptre, A. & Williamson, E. A. *Pan troglodytes*. *The IUCN Red List of Threatened Species*, e.T15933A129038584 (2016).

220 Bauer, H., Packer, C., Funston, P. F., Henschel, P. & Nowell, K. *Panthera leo*. *The IUCN Red List of Threatened Species*, e.T15951A115130419 (2016).

221 Quigley, H. *et al*. *Panthera onca*. *The IUCN Red List of Threatened Species* e.T15953A123791436 (2017).

222 Stein, A. B. *et al*. *Panthera pardus*. *The IUCN Red List of Threatened Species* e.T15954A102421779 (2016).

223 Goodrich, J. *et al*. *Panthera tigris*. *The IUCN Red List of Threatened Species,* e.T15955A50659951 (2015).

224 McCarthy, T., Mallon, D., Jackson, R., Zahler, P. & McCarthy, K. *Panthera uncia*. *The IUCN Red List of Threatened Species*, e.T22732A50664030 (2017).

225 Kingdon, J., Butynski, T. M. & De Jong, Y. *Papio cynocephalus*. *The IUCN Red List of Threatened Species*, e.T92250442A92250811 (2016).

226 Gippoliti, S. & Ehardt, T. *Papio hamadryas*. *The IUCN Red List of Threatened Species,* e.T16019A5354647 (2008).

227 Gongora, J. *et al*. *Pecari tajacu*. *The IUCN Red List of Threatened Species,* e.T41777A10562361 (2011).

228 Oates, J. F. *et al*. *Perodicticus potto*. *The IUCN Red List of Threatened Species* e.T91995408A91995190 (2016).

229 Woinarski, J. & Burbidge, A. A. *Phascolarctos cinereus*. *The IUCN Red List of Threatened Species* e.T16892A21960344 (2016).

230 D'elia, G. *Phyllotis darwini*. *The IUCN Red List of Threatened Species,* e.T17224A22341324 (2016).

231 Ancrenaz, M. *et al*. *Pongo pygmaeus*. *The IUCN Red List of Threatened Species*, e.T17975A123809220 (2016).

232 Reyna, R., Jori, F., Querouil, S. & Leus, K. *Potamochoerus porcus*. *The IUCN Red List of Threatened Species* e.T41771A100469961 (2016).
233 Helgen, K., Kays, R. & Schipper, J. *Potos flavus*. *The IUCN Red List of Threatened Species,* e.T41679A45215631 (2016).
234 Timm, R., Cuarón, A. D., Reid, F., Helgen, K. & González-Maya, J. F. *Procyon lotor*. *The IUCN Red List of Threatened Species* e.T41686A45216638 (2016).
235 Nielsen, C., Thompson, D., Kelly, M. & Lopez-Gonzalez, C. A. *Puma concolor*. *The IUCN Red List of Threatened Species* e.T18868A97216466 (2015).
236 Gunn, A. *Rangifer tarandus*. *The IUCN Red List of Threatened Species,* e.T29742A22167140 (2016).
237 Duckworth, J. W., Kumar, N. S., Pokharel, C. P., Sagar Baral, H. & Timmins, R. *Rucervus duvaucelii*. *The IUCN Red List of Threatened Species,* e.T4257A22167675 (2015).
238 Savage, A. & Causado, J. *Saguinus oedipus*. *The IUCN Red List of Threatened Species* e.T19823A17930260 (2014).
239 Boubli, J.-P., Rylands, A. B., de la Torre, S. & Stevenson, P. *Saimiri sciureus*. *The IUCN Red List of Threatened Species*, e.T41537A10494364 (2008).
240 Mitra, S. & Molur, S. *Semnopithecus entellus*. *The IUCN Red List of Threatened Species* e.T39832A10274074 (2008).
241 Cassola, F. *Sigmodon hispidus*. *The IUCN Red List of Threatened Species*, e.T20213A115157685 (2016).
242 DeMatteo, K., Michalski , F. & Leite-Pitman, M. R. P. *Speothos venaticus*. *The IUCN Red List of Threatened Species,* e.T20468A9203243 (2011).
243 Jordan, N. R. & Do Linh San, E. *Suricata suricatta*. *The IUCN Red List of Threatened Species*, e.T41624A45209377 (2015).
244 Oliver, W. & Leus, K. *Sus scrofa*. *The IUCN Red List of Threatened Species* e.T41775A10559847 (2008).
245 Nijman, V. & Geissman, T. *Symphalangus syndactylus*. *The IUCN Red List of Threatened Species* e.T39779A10266335 (2008).
246 Group, I. S. A. S. *Syncerus caffer*. *The IUCN Red List of Threatened Species,* e.T21251A50195031 (2008).

247 Tsytsulina, K., Formozov, N., Shar, S., Lkhagvasuren, D. & Sheftel, B. *Tamias sibiricus*. *The IUCN Red List of Threatened Species* e.T21360A115161465 (2016).

248 Cassola, F. *Tamias striatus*. *The IUCN Red List of Threatened Species*, e.T42583A115191543 (2016).

249 Traeholt, C. *et al*. *Tapirus indicus*. *The IUCN Red List of Threatened Species* e.T21472A45173636 (2016).

250 Traeholt, C. *et al*. *Tapirus indicus*. *The IUCN Red List of Threatened Species* e.T21472A45173636 (2016).

251 Keuroghlian, A. *et al*. *Tayassu pecari*. *The IUCN Red List of Threatened Species*, e.T41778A44051115 (2013).

252 Gippoliti, S. & Hunter, C. *Theropithecus gelada*. *The IUCN Red List of Threatened Species,* e.T21744A9316114 (2008).

253 Solari, S. & Palma, E. *Thylamys elegans*. *The IUCN Red List of Threatened Species* e.T40517A22172461 (2016).

254 Velez-Liendo, X. & García-Rangel, S. *Tremarctos ornatus*. *The IUCN Red List of Threatened Species* e.T22066A123792952 (2017).

255 Sargis, E. & Kennerley, R. *Tupaia glis*. *The IUCN Red List of Threatened Species* e.T111872341A123796056 (2017).

256 McLellan, B. N., Proctor, M. F., Huber, D. & Michel, S. *Ursus arctos*. *The IUCN Red List of Threatened Species* e.T41688A121229971 (2017).

257 Wiig, Ø. *et al*. *Ursus maritimus*. *The IUCN Red List of Threatened Species* e.T22823A14871490 (2015).

258 Andriaholinirina, N. *et al*. *Varecia rubra*. *The IUCN Red List of Threatened Species*, e.T22920A16121712 (2014).

259 Andriaholinirina, N. *et al*. *Varecia variegata*. *The IUCN Red List of Threatened Species*, e.T22918A16121857 (2014).

260 Acebes, P. *et al*. *Vicugna vicugna*. *The IUCN Red List of Threatened Species*, e.T22956A145360542 (2018).

261 Murdoch, J. D. *Vulpes corsac*. *The IUCN Red List of Threatened Species* e.T23051A59049446 (2014).

262 Angerbjörn, A. & Tannerfeldt, M. *Vulpes lagopus*. *The IUCN Red List of Threatened Species*, e.T899A57549321 (2014).

263 Hoffmann, M. & Sillero-Zubiri, C. *Vulpes vulpes*. *The IUCN Red List of Threatened Species*, e.T23062A46190249 (2016).
