# Supplementary material for: Coevolution of vocal signal characteristics and hearing sensitivity in forest mammals
Source: Nat Commun. 2019 Jun 25;10:2778. doi: 10.1038/s41467-019-10768-y (PMC6592901; doi:10.1038/s41467-019-10768-y)
Supplement: Supplementary file 3 — Reporting Summary [file 41467_2019_10768_MOESM3_ESM.pdf]

nature research

Corresponding author(s): Benjamin D. Charlton

Last updated by author(s): May 20, 2019

Reporting Summary

Nature Research wishes to improve the reproducibility of the work that we publish. This form provides structure for consistency and transparency in reporting. For further information on Nature Research policies, see Authors & Referees and the Editorial Policy Checklist.

Statistics

For all statistical analyses, confirm that the following items are present in the figure legend, table legend, main text, or Methods section.

n/a	Confirmed	
☐	☒	The exact sample size (n) for each experimental group/condition, given as a discrete number and unit of measurement
☒	☐	A statement on whether measurements were taken from distinct samples or whether the same sample was measured repeatedly
☐	☒	The statistical test(s) used AND whether they are one- or two-sided<br>*Only common tests should be described solely by name; describe more complex techniques in the Methods section.*
☐	☒	A description of all covariates tested
☐	☒	A description of any assumptions or corrections, such as tests of normality and adjustment for multiple comparisons
☐	☒	A full description of the statistical parameters including central tendency (e.g. means) or other basic estimates (e.g. regression coefficient) AND variation (e.g. standard deviation) or associated estimates of uncertainty (e.g. confidence intervals)
☐	☒	For null hypothesis testing, the test statistic (e.g. F, t, r) with confidence intervals, effect sizes, degrees of freedom and P value noted<br>*Give P values as exact values whenever suitable.*
☐	☒	For Bayesian analysis, information on the choice of priors and Markov chain Monte Carlo settings
☒	☐	For hierarchical and complex designs, identification of the appropriate level for tests and full reporting of outcomes
☐	☒	Estimates of effect sizes (e.g. Cohen's d, Pearson's r), indicating how they were calculated

Our web collection on statistics for biologists contains articles on many of the points above.

Software and code

Policy information about availability of computer code

Data collection	No software was used to collect the data.
Data analysis	R version 3.2.3 was used to analyze the data. Praat version 6.0.31 was used to conduct the acoustic analysis .

For manuscripts utilizing custom algorithms or software that are central to the research but not yet described in published literature, software must be made available to editors/reviewers. We strongly encourage code deposition in a community repository (e.g. GitHub). See the Nature Research guidelines for submitting code & software for further information.

Data

Policy information about availability of data

All manuscripts must include a data availability statement. This statement should provide the following information, where applicable:

- Accession codes, unique identifiers, or web links for publicly available datasets
- A list of figures that have associated raw data
- A description of any restrictions on data availability

The data that support the findings of this study are available in the Supplementary Information.

Field-specific reporting

Please select the one below that is the best fit for your research. If you are not sure, read the appropriate sections before making your selection.

☐ Life sciences ☐ Behavioural & social sciences ☒ Ecological, evolutionary & environmental sciences

For a reference copy of the document with all sections, see nature.com/documents/nr-reporting-summary-flat.pdf

Ecological, evolutionary & environmental sciences study design

All studies must disclose on these points even when the disclosure is negative.

Study description	*Briefly describe the study. For quantitative data include treatment factors and interactions, design structure (e.g. factorial, nested, hierarchical), nature and number of experimental units and replicates.*
Research sample	The research samples were data from published studies or acoustic data extracted from audio recordings. The original audio recordings had a sampling rate of 44.1 kHz or 48 kHz and an amplitude resolution of 16 bits. The data were supposed to represent forest mammals and other terrestrial mammal species.
Sampling strategy	We collected data from as many species as possible.
Data collection	Audiogram data was collected from the literature. The audio recordings were downloaded from the Animal Sound Archive at the Museum für Naturkunde Berlin (http://www.animalsoundarchive.org/) and the Macaulay library at the Cornell Lab of Ornithology (https://www.macaulaylibrary.org), or extracted from commercially available audio CDs. BDC provided audio recordings for an additional six species. The data was collected by BDC. Some of the audiogram data was collected by MO
Timing and spatial scale	The data was collected over six month period, May-Nov 2018.
Data exclusions	We collected audio recordings from captive animals to avoid examining vocalisations that had already been degraded by environmental transmission in a given species typical habitat. In addition, only adult vocalisations or audiogram data were collected.
Reproducibility	We provide the data used to generate the results as supplementary material. The R code is available from the first and corresponding author (BDC) on request. All other information to repeat the analysis is provided in the methods section.
Randomization	Data was organized into forest-living versus other terrestrial mammals. Covariates known to affect hearing sensitivity (head size) and acoustic structure (body mass) were entered into the statistical models.
Blinding	Blinding was not relevant to this comparative study. The audiogram data is published and the acosutic analysis of recordings was conducted using automated scripts (in Praat) i.e. no subjective analysis was conducted.

Did the study involve field work? ☐ Yes ☒ No

Reporting for specific materials, systems and methods

We require information from authors about some types of materials, experimental systems and methods used in many studies. Here, indicate whether each material, system or method listed is relevant to your study. If you are not sure if a list item applies to your research, read the appropriate section before selecting a response.

Materials & experimental systems

n/a	Involved in the study	
☒	☐	Antibodies
☒	☐	Eukaryotic cell lines
☒	☐	Palaeontology
☒	☐	Animals and other organisms
☒	☐	Human research participants
☒	☐	Clinical data

Methods

n/a	Involved in the study	
☒	☐	ChIP-seq
☒	☐	Flow cytometry
☒	☐	MRI-based neuroimaging
